# Understanding User Intent in Code-Mixed Sexual and Reproductive Health Queries in Urban India: Hierarchical Classification Approach Using Large Language Models

**DOI:** 10.2196/86545

**Published:** 2026-03-24

**Authors:** Sumon Kanti Dey, Manvi S, Aradhana Thapa, Meet Shah, Zeel Mehta, Shraddha Kale Kapile, Tanvi Divate, Suhani Jalota, Azra Ismail

**Affiliations:** 1 Department of Biomedical Informatics, School of Medicine, Emory University, 101 Woodruff Circle, Atlanta, GA, 30322, United States, 1 4046437205; 2 Hubert Department of Global Health, Rollins School of Public Health, Emory University, Atlanta, GA, United States; 3 Myna Mahila Foundation, Mumbai, India; 4 Hoover Institution, Stanford University, Stanford, CA, United States

**Keywords:** sexual and reproductive health, large language models, code-mixing, Hinglish, hierarchical classification, conversational agents

## Abstract

**Background:**

Sexual and reproductive health (SRH) remains a stigmatized and taboo topic globally, limiting access to reliable information. These challenges are heightened in the Global South, where linguistic and cultural diversity further complicates information access. In India (the study context), many individuals express SRH concerns in code-mixed language, such as Hinglish (code-mixed Hindi and English), and use colloquial terms. Large language models (LLMs) could help answer SRH questions, but most are trained for English and may perform poorly on code-mixed text and miss cultural nuances. Our research aims to address this gap by assessing the current state of LLMs in understanding user intent in SRH queries for a low-resource language.

**Objective:**

We evaluate the effectiveness of proprietary, multilingual open-weight, and Indic LLMs in zero-shot settings for identifying user intent in code-mixed Hinglish SRH queries. Our goal is to assess how well LLMs assign correct labels in a 2-level hierarchical classification (topic and subtopic). We take a hierarchical approach because SRH queries are complex and context-dependent; flat labels may obscure clinically important distinctions and lead to misdirected guidance. We also characterize common error types driving misclassification.

**Methods:**

We analyzed 4161 deidentified questions about SRH in Hinglish, collected by our partner nonprofit organization (Myna Mahila Foundation) in an underserved community in urban Mumbai. Queries were annotated into 8 topics and 40 subtopics using a hierarchical framework that captured linguistic, cultural, and contextual variation. We evaluated proprietary, multilingual open-weight, and Indic-specific LLMs in zero-shot settings. Performance was measured using hierarchical *F*_1_ (h*F*_1_), Exact Match, and topic- and subtopic-level accuracy.

**Results:**

Proprietary models achieved the strongest results, with GPT-5 performing best overall (h*F*_1_= 0.784). Among open-weight systems, Sarvam-M emerged as the top-performing Indic model (h*F*_1_=0.757), ranking just below the top-performing proprietary model and performing comparably with Claude-3.5-Sonnet (0.745; Anthropic) as well as large multilingual systems such as Llama-3.3-70B-Instruct (0.742; Meta) and Gemma-3-27B-IT (0.739; Google). Other Indic models performed considerably lower (eg, Llama-3-Gaja-Hindi-8B [0.596; CognitiveLab], Krutrim-2-Instruct [0.558; OLA Krutrim Team], and Airavata [0.404; AI4Bharat]). Smaller multilingual open-weight models, including Mixtral-8 × 7B-Instruct (0.593), Llama-3.1-8B-Instruct (0.630), Gemma-2-9B-IT (0.657), consistently outperformed them, showing that parameter size alone does not explain performance gaps. While models generally captured broad topical intent, they frequently failed at fine-grained intent recognition, especially with euphemisms, colloquial expressions, and locally or culturally situated questions.

**Conclusions:**

Hierarchical classification revealed persistent gaps in how LLMs handle code-mixed queries. Proprietary models performed best, but Sarvam-M shows that open-weight Indic systems can achieve performance near state-of-the-art models when supported by robust training data, cultural adaptation, and appropriate scale. These findings highlight the potential of localized, culturally aligned models to advance linguistically inclusive artificial intelligence tools and expand equitable access to SRH information in underserved populations globally.

## Introduction

### Background

Recent advancements in large language models (LLMs) present an opportunity to address significant gaps in health care information delivery. LLMs could be leveraged to simplify complex medical information, respond to patient queries, and enhance health literacy among the general population [1,2]. Despite these advancements, there remain significant disparities in the performance of these models across languages, especially in health care tasks [3]. Most LLMs are predominantly centered on the English language [4,5]. They can fail to recognize local dialects, cultural nuances, and speaking patterns, especially for non-English speaking populations that are less represented online. Health communication is further shaped by social dynamics, including gender, educational status, functional literacy, and cultural context [6-8]. Our research focuses on addressing these challenges in the context of India by evaluating the performance of LLMs in detecting user intent in the context of sexual and reproductive health (SRH).

SRH presents unique challenges for health information delivery, given that stigma, misinformation, and social barriers can restrict individuals—especially women—from seeking reliable health information and medical support [9,10]. For instance, 78% of the 15 million abortions in India take place outside medical facilities, highlighting the need for better access to reproductive health services and information [11]. Deep-rooted societal taboos also prevent open discussions about sex-related topics, exacerbating barriers to SRH awareness and services [12]. At the same time, SRH is a time-sensitive domain in which delays can have serious consequences. In India, lack of early pregnancy care leads to undetected and unmanaged conditions such as anemia, diabetes, hypertension, and infections, which are significant causes of pregnancy loss and maternal mortality [13,14].

While a multitude of online platforms exist where individuals can engage with health care professionals or even community members (eg, Reddit; Reddit, Inc), the reach of these platforms remains low in underserved communities in India. The absence of such inclusive platforms for linguistically diverse, resource-constrained communities further contributes to health inequities, leaving many women without access to critical SRH-related knowledge and services. Prior work by Wang et al [15] has shown the potential of a rule-based conversational chatbot for SRH support among young people in India (SnehAI), demonstrating strong user engagement and information-seeking behavior. We build on this work by evaluating the potential of LLMs to support the understanding of user intent in code-mixed SRH queries for such interventions.

Adding to these challenges is the extensive linguistic diversity within India, which significantly influences communication patterns, particularly in informal and colloquial settings. A notable linguistic phenomenon prevalent across India is code-mixing, the practice of blending multiple languages within a single conversation or utterance. We focus on Hinglish—a popular form of code-mixing involving Hindi and English, where individuals use the phonetic Latin script instead of the Devanagari script to write Hindi words [16]. This was often the preferred mode of typing for the population on which we focus in this study and has been documented in prior research in other Hindi-speaking populations in India [17,18]. Code-mixing remains a long-standing challenge in natural language understanding research, with several publicly available LLMs still struggling to interpret and generate code-switched text [19,20].

**Figure 1.**
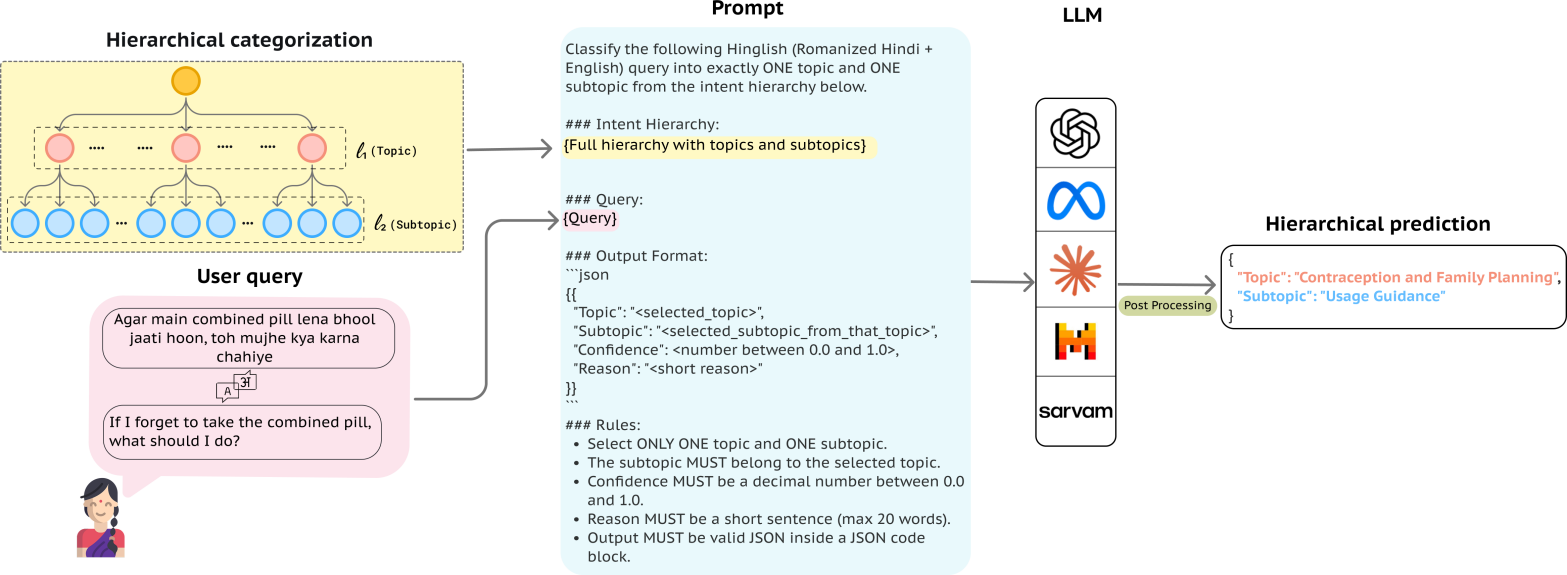
**Hierarchical sexual and reproductive health (SRH) intent classification framework**. This figure illustrates our approach to processing a code-mixed SRH user query (an English translation of the original Hinglish text is shown for clarity) using a large language model (LLM) to infer intent. The model maps the query to both a topic and its corresponding subtopic, producing structured intent classification. JSON: JavaScript Object Notation.

Another significant challenge is the inherently layered and context-dependent nature of questions that individuals frequently ask about SRH. Broader concerns related to pregnancy may branch into distinct subtopics such as antepartum emergency, postpartum pain, infertility, or abortion, reflecting the complex structure of real-world health inquiries. Traditional flat classification approaches may collapse these distinctions [21], leading to the generation of misleading responses that may fail to capture critical aspects of care and support. Our research aims to address this gap by taking a hierarchical classification approach to understanding user intent in SRH queries (Figure 1).

In summary, this study contributes the following: (1) we designed a hierarchical intent structure for SRH queries with 2 levels (topic and subtopic) to capture user intent in code-mixed Hinglish, providing clear label descriptions to support consistent annotation and evaluation, (2) we evaluated several state-of-the-art proprietary and open-weight multilingual LLMs (including Indic LLMs) to measure how effectively they handle hierarchical intent classification of SRH-related queries expressed in Hinglish. This evaluation also provides insight into how well LLMs can handle code-mixed text, ensuring broader applicability in the real-world health care contexts, and (3) we conducted a qualitative error analysis with thematic coding across models to examine misclassifications in code-mixed SRH queries. We explain why these errors occur and how models misread culturally sensitive terms and euphemisms. Representative annotated examples from the dataset are provided in Table 1.

**Table 1.** Sample annotated user queries from the dataset. The examples below present both transliteration and code-mixing between Hindi and English.

Query	English translation	Topic	Subtopic
Periods agar monthly na aaye to kya karen?	What should I do if my periods are not regular every month?	Menstrual Health	Menstrual Cycle Information
Gabhnirodhak goliyan lene se pehle mujhe doctor se kya puchna chahiye?	What should I ask the doctor before taking birth control pills?	Contraception and Family Planning	Usage Guidance
Agar PCOD^a^ ka 3 mahina dawa chalane ke baad bhi shi nahi hua to kya kare?	If PCOD^a^ does not improve after three months of medication, what should I do?	PCOS^b^ or PCOD^a^	Management
Agar abhi bacha nahi rakhna hai aur fir bhi pregnant ho gaye to abortion ke liye kya kare?	If I do not want to have a baby right now but still get pregnant, what should I do for an abortion?	Pregnancy and PNC^c^	Abortion

a PCOD: polycystic ovarian disease.

b PCOS: polycystic ovary syndrome.

c PNC: postnatal care.

### Related Work

Hierarchical classification is a method of assigning items to categories organized within a hierarchy of classes [22,23]. This approach has been broadly used in a range of domains where layered structures are important, including e-commerce user query categorization [24,25], health care question answering [26,27], clinical guideline classification [21], and personalized health care analysis of women’s menstrual health disorders [28]. Its advantages lie in its ability to model the inherent complexity of user intent, particularly in health care contexts where individuals begin their health care concerns by first referencing broader domains and then subsequently focusing on specific details or contexts. While flat classification systems are widely used as classification baselines, their lack of hierarchical awareness limits their effectiveness, especially in domains where understanding class structure is essential [21,29]. Although hierarchical classification has demonstrated clear benefits across diverse applications, its potential remains underexplored in the context of code-mixed languages and SRH, where understanding layered and implicit user intent is especially important.

The recent development of highly parameterized language models, such as GPT-5 (OpenAI) [30], GPT-4o (OpenAI) [31], Llama 3 (Meta) [32], Gemma 3 (Google) [33], among many others, has significantly enhanced health care reasoning, understanding, and summarization [34]. These models can generate concise and user-friendly summaries and have the potential to make medical information more accessible to the general public. Despite these developments, a major limitation remains their reliance on training data in English, making them less effective for regional languages [12,35]. For instance, Llama 3 sources 90% of its pretraining data from English sources [4], while a substantial portion of GPT-4’s pretraining data is similarly English-dominant [5]. This linguistic bias creates information disparities across languages, where equivalent questions in different languages may produce inconsistent and inaccurate responses [36]. Our study seeks to address this gap.

Recent research has increasingly focused on using chatbots to provide health care assistance, particularly around SRH. For example, Wang et al [15] designed an SRH-focused rule-based chatbot on Facebook Messenger (Meta Platforms, Inc) to analyze how users engage with artificial intelligence (AI) to seek health information. Their study found that users frequently shared personal concerns and SRH-related queries in code-mixed languages, highlighting the need for linguistically adaptable health care AI models. There are several reasons why LLMs can be useful for SRH information delivery, including gaps in sexual education [37] and societal stigma and taboo around sex-related topics [12]. However, recent studies indicate that LLMs struggle with Indic languages, particularly when queries are code-mixed and culturally situated [38]. To analyze these issues systematically, we collected a dataset called sexual and reproductive health queries (SRHQ)-India, which captures real-world Indian SRH user queries in a code-mixed format.

As queries in SRH domains are often linguistically and contextually diverse, the hierarchical categorization approach offers the potential for more accurate and interpretable results. Recent advancements in LLMs have made significant improvements in flat text classification, especially in the health care domain. In our work, we seek to evaluate LLMs’ understanding of the hierarchical classification task. In this regard, we explore the use of a zero-shot learning technique that involves crafting prompts to enable language models to generate useful responses without prior examples, relying entirely on pretrained knowledge to tackle the new task. This technique has been used in several health care applications, such as capturing the context of clinical text [39] and classifying health care queries [40]. In our work, we leverage zero-shot learning to evaluate the efficiency of language models in processing code-mixed user queries.

## Methods

### Overview

In this study, we used a dataset gathered by the Myna Mahila Foundation, a nonprofit women’s health organization in India. The data were collected through a preliminary SRH chatbot prototype that was developed and piloted with 488 women in informal settlements of Mumbai, India, from October 2023 to December 2024 to capture real-world user queries on SRH, including both text and voice input. This is part of their ongoing effort to improve access to SRH information for women and girls in urban slums. Below in “Dataset Generation and Curation” and “Data Annotation” subheadings, we detail our approach to structuring and annotating the dataset. We also outline our evaluation framework for model performance.

### Dataset Generation and Curation

The dataset originally contained 4858 queries and was refined to reduce redundancy using cosine similarity computed over term frequency–inverse document frequency representations of the text. Queries were first normalized using standard text preprocessing, including Unicode correction, lowercasing, whitespace normalization, and removal of basic punctuation. Cosine similarity was then used to identify syntactically similar queries, and pairs with a similarity score of 90% or higher were considered near duplicates, and only one was retained. For example, *“*kya saheli tablet lene se periods ka date badal jata hai?*”* or “saheli tablet se periods ka date badal jata hai kya*”* convey the same meaning: “Does taking the Saheli tablet (a nonhormonal contraceptive pill) change the period date?” Term frequency–inverse document frequency–based lexical similarity was chosen instead of pretrained sentence embeddings because many widely used embedding models are trained predominantly on monolingual or English-centric data and perform poorly on code-mixed Indian languages [41,42]. Moreover, our aim was to remove lexically rephrased queries rather than infer semantic equivalence. In addition, we removed queries containing fewer than three words to exclude vague or incomplete inputs. We also filtered out greeting-based queries, such as *“*Namaste*”* or “Aap kaise hai,” which means “How are you,” which do not contribute to intent classification.

After filtering, we obtained a final dataset of 4161 queries, primarily in Hinglish. The dataset consists of queries with misspelled English words, transliterated Hindi terms, and borrowed English medical words that are common in everyday SRH talk (eg, periods, condom, pregnancy, in-vitro fertilization [IVF], and polycystic ovary syndrome). We also observed queries related to cultural practices or religious beliefs. For example, “Masik pali aane se Bhagvan ke pas kau nahi jana chahiye?” which in English translates to “Why should one avoid going to god or temples during periods?” This query centers around the religious norm of how women are advised not to visit the temple during their periods in certain cultures and communities in India. Additionally, the query *“*Det ke pahele piryat q aata hai*”* translates to “Why periods come before the date?” where det (date) and piryat (period) are misspelled terms. Such variations highlight the real-world linguistic challenges in SRH-related conversations and underscore the need for models capable of accurately processing noisy, code-mixed, and culturally or religiously influenced queries.

Beyond linguistic complexities, the dataset also captures deeply ingrained cultural myths and misinformation, particularly related to pregnancy and gender beliefs. For instance, the query *“*Main pregnant hu or jinko sirf ladkiya hai kya unko dekte rahne se kya muje ladki hogi?*”* translates to “I am pregnant, if I keep seeing women who have only given birth to daughters, will I also have a daughter?” Similarly, *“*Kya muje sirf ladka hi chahiye to uske liye khuch upay hai kya?*”* means “Is there any way to have only a boy?” These queries reflect long-standing cultural expectations in some communities in India, where socioeconomic structures reinforce a preference for sons. Such myths not only influence reproductive decisions but also contribute to gender-based discrimination and misinformation.

We adopted a hierarchical thematic structure for annotation. Queries were first classified into broad topics and then further refined into subcategories to capture the complexity of SRH-related concerns. For example, questions related to postpartum, antepartum, and abortion fall under the broad category of Pregnancy and Postnatal Care (PNC) category, while questions related to the Menstrual Health category are further classified based on their context to subtopics, including Menstrual Cycle Information, Menstrual Flow, or Period Pain Management. We have also included Mental Health and Wellness, with subtopics including Stress Management and Safety Concerns, because stress, sleep, mood, and anxiety issues often co-occur with SRH questions and influence help-seeking and guidance needs. Placing these queries within the SRH hierarchy keeps related concerns together and improves intent interpretation. Table 2 provides the annotation framework in detail. The annotation guidelines were developed through an iterative process of feedback from doctors and program staff at the Myna Mahila Foundation. This helped standardize interpretations and improve annotation reliability across the dataset. Key descriptive statistics of the dataset are provided in Table 3. In addition, we quantify the degree of intrasentential Hindi-English code-mixing using the Code-Mixing Index (CMI); dataset-level CMI statistics are reported in Table 3, and the metric is described in the following “CMI Calculation” subheading.

**Table 2.** Annotated dataset distribution by topic and subtopic.

Topic and subtopic	Queries (N=4161), n (%)
Contraception and Family Planning	1417 (34.1)
Family Planning Queries	522 (36.8)
Usage Guidance	291 (20.5)
Types of Contraceptives	209 (14.7)
Sterilization	184 (13)
Effectiveness and Duration	127 (9)
Side Effects	84 (5.9)
Menstrual Health	1104 (26.5)
Menstrual Cycle Information	673 (61)
Period Pain Management	325 (29.4)
Sanitary Products and Hygiene	62 (5.6)
Menstrual Flow	44 (4)
Pregnancy and PNC^a^	547 (13.1)
Pregnancy Information	290 (53)
Antepartum	120 (21.9)
Infertility	53 (9.7)
Abortion	26 (4.8)
Postpartum	23 (4.2)
Miscarriage	20 (3.7)
Breastfeeding	15 (2.7)
Sexual and Vaginal Health	322 (7.7)
Sex-Related Queries	128 (39.8)
Vaginal Health and Discharge	127 (39.4)
Reproductive Anatomy	26 (8.1)
Urinary Tract Infections (UTI)	16 (5)
Sexually Transmitted Infections (STI or STD)	15 (4.7)
Vaginal or Uterine Infections	10 (3.1)
PCOS or PCOD^b^^c^	101 (2.4)
Information	49 (48.5)
Management	28 (27.7)
Symptoms	24 (23.8)
HIV^d^	52 (1.2)
Stigma and Awareness	14 (26.9)
Treatment	14 (26.9)
Prevention	12 (23.1)
Symptoms and Early Detection	12 (23.1)
Mental Health and Wellness	22 (0.5)
Stress Management	13 (59.1)
Information and Safety Concerns	9 (40.9)
Other	596 (14.3)
General Health Queries	361 (60.6)
Diet and Nutrition	97 (16.3)
Exercise and Fitness	46 (7.7)
Health Equity and Access	38 (6.4)
Marriage and Relationships	21 (3.5)
Misconceptions and Myths	13 (2.2)
Child Health	12 (2)
Cultural, Religious, or Moral Norms	8 (1.3)

a PNC: postnatal care.

b PCOS: polycystic ovary syndrome.

c PCOD: polycystic ovarian disease.

d HIV: human immunodeficiency virus.

**Table 3.** Statistics of the dataset.

Statistic	Values
Total number of topics	8
Total number of subtopics	40
Total number of queries	4161
Maximum question length (words)	71 words
Average question length (words)	10.56 words
% queries in transliterated Hindi^a^	61.53
% queries in intrasentential mixing^b^	38.37
Code-Mixing Index (CMI), %, mean (SD)	37.38 (10.43)

a Transliterated Hindi refers to text written in Hindi using the Latin script, often incorporating English loan words common in sexual and reproductive health (SRH) contexts (eg, periods, condom, and pregnancy).

b Intrasentential mixing refers to text where Hindi and English elements are combined within the same sentence, written in Latin script.

Our approach to annotation serves 2 purposes. First, hierarchical classification offers a deeper perspective on SRH concerns, allowing the identification of common themes and trends in user queries. It strengthens decision support for stakeholders, including health care professionals, policymakers, and organizations, by offering a well-organized dataset that can inform better resource allocation and intervention strategies. Second, hierarchical classification can enhance user guidance and response accuracy based on user intent, ensuring that individuals are directed to relevant and specific information tailored to their concerns.

### CMI Calculation

The CMI [43] is an utterance-level, ratio-based metric that quantifies intrasentential code-mixing by measuring the proportion of lexical tokens that do not belong to the matrix (dominant) language.

CMI is defined as:


$$\text{CMI}=100\times \left(1-\frac{\max\left(w_{i}\right)}{n-u}\right),\text{if }n>u$$

Where n is the total number of tokens, u is the number of language-independent tokens, n-u is the sum of the number of tokens from N languages, and $\max\{w_{i}\}$is the highest number of words belonging to a particular language. If an utterance contains only language-independent tokens (n=u), the CMI is defined as zero.

We used the pretrained FastText (Meta AI) language identification model, which supports 176 languages [44], to automatically assign language labels at the token level. FastText has been widely used for language identification in multilingual and code-mixed text due to its efficiency and robustness on short lexical units, and its use of subword information [45,46]. Most queries in the dataset are bilingual in nature, with one dominant language, either English or Romanized Hindi, serving as the matrix language within each utterance. We used the model to identify English tokens (and assumed the remaining tokens to be Hindi) due to potential concerns about its performance on Hindi words. Numerals, punctuation, and symbols were treated as language-independent. This automated procedure enabled consistent computation of sentence-level CMI scores across all 4161 queries.

### Data Annotation

We annotated the user queries into 8 categories, which were further divided into subcategories to capture the depth and complexity of the topics. The annotation of our code-mixed dataset on SRH required both strong linguistic competence and domain expertise. Since the data are code-mixed (Hindi and English), they present unique represents unique challenges such as nonstandard spelling, switching between languages in the same query, and the cultural nuances embedded in users’ expressions. To meet these complexities, this study included 2 experienced annotators with backgrounds in public health and global health. Both annotators are native or fluent speakers of Indian languages and coauthors of this paper, ensuring familiarity with cultural and linguistic contexts relevant to SRH queries. Both were guided by a comprehensive annotation manual, refined by subject matter experts (public health care professionals and medical doctors). Some user questions could overlap with multiple topics or subtopics (for instance, a question about convincing a partner to use contraceptives could fall under the “Family Planning” or “Marriage and Relationship” category). In such cases, annotators selected the closest-fitting category. The full annotation guidelines are provided in Multimedia Appendix 1.

To ensure annotation quality and consistency, we adopted an iterative annotation process. Initially, 10% of the dataset was annotated independently by both annotators. This step was particularly critical, as it allowed us to refine annotation guidelines and address challenges arising from orthographic variations, code-mixing, and semantic ambiguities. Discrepancies were resolved through discussion and led to refinements in the annotation guidelines. We continued the iterative co-annotation process until the annotators consistently achieved a 95% agreement threshold during the pilot phase. Once the threshold was met, the remaining data were divided between the 2 annotators for independent annotation. We used Cohen kappa [47] across overlapping annotated samples to compute interannotator agreement. The resulting score of 83% indicated substantial agreement, reflecting almost perfect agreement [48]. The distribution of the query categories and subcategories is provided in Table 2.

### Evaluation Metrics

To evaluate model performance on our hierarchical classification task, we adopted the Hierarchical *F*_1_-score (h*F*_1_) proposed by Kosmopoulos et al [23]. It extends traditional metrics by explicitly considering the hierarchical relationship among labels. To compute this metric, we augment both the predicted labels ($\hat{Y}$) and the ground truth labels (Y) by incorporating their ancestor labels from the hierarchy, resulting in augmented sets $\hat{Y}aug$ *and* Yaug. This augmentation ensures that the evaluation accurately reflects hierarchical dependencies between topics and subtopics.

The hierarchical *F*_1_-score (h*F*_1_) is defined as:


(1) $$hF_{1}=\frac{2\cdot hPr\cdot hRe}{hPr+hRe},\text{ where }hPr=\frac{\sum_{i}|\hat{Y_{aug}}\cap Y_{aug}|}{\sum_{i}|\hat{Y_{aug}}|},hRe=\frac{\sum_{i}|\hat{Y_{aug}}\cap Y_{aug}|}{\sum_{i}|Y_{aug}|}$$

Here, hPr and hRe (hierarchical precision and recall) evaluate the proportion of correctly predicted hierarchical labels among all predicted and actual labels, respectively. Additionally, we used supporting metrics, including Exact Match, which evaluates the percentage of queries where both the topic and subtopic predictions exactly match the ground truth labels. Accuracy@l_1_ measures the topic-level accuracy, reflecting the proportion of samples where the predicted topic is correct. Accuracy@l_2_ measures conditional subtopic accuracy, defined as the proportion of correct subtopic predictions given that the topic was correctly predicted. These complementary metrics collectively provide a comprehensive evaluation of the model’s performance, capturing strict end-to-end correctness (Exact Match)*,* coarse-grained topic identification (Accuracy@l_1_), fine-grained subtopic discrimination (Accuracy@l_2_), and hierarchical sensitivity (h*F*_1_).

### Experimental Analysis

In this study, we evaluate the performance of language models on a hierarchical classification task in a zero-shot setting, where the model attempts to generalize output patterns at the topic and subtopic levels for data it has not encountered during training. Zero-shot evaluation is particularly challenging because it requires models to infer patterns without explicit task-specific fine-tuning.

In our setup, models were provided with the hierarchical structure and the list of possible topic and subtopic labels, but not with the detailed semantic definitions used in the human annotation guideline. This was a deliberate design choice to evaluate the models’ intrinsic ability to interpret real-world, code-mixed SRH queries without additional semantic instruction. It also reflects practical deployment scenarios where the model must generalize from raw user input rather than rely on curated taxonomies at the time of inference.

Our evaluation includes 7 open-weight multilingual models, 5 Indic-specific models, and 3 proprietary models. The open-weight multilingual models are Mixtral-8 × 7B-Instruct (Mistral AI) [49], Llama-3.1-8B-Instruct (Meta) [32], Llama-3.3-70B-Instruct (Meta), Gemma-2-9B-IT (Google) [50], Gemma-3-27B-IT (Google) [33], Qwen-2.5-7B-Instruct (Alibaba Group) [51], and Aya-Expanse-8B (Cohere Labs) [52]. The technical reports of these models demonstrate their multilingual capabilities on benchmark datasets, making them strong candidates for handling code-mixed data.

The Indic models are Airavata [53], an instruction-tuned Hindi language model developed by AI4Bharat; AryaBhatta-GemmaGenZ-Vikas-Merged [54] (referred to as AryaBhatta [GenVR Research]), a model trained on 9 Indic languages that excels particularly in Hindi reasoning and literature tasks; and Llama-3-Gaja-Hindi-8B [55], a bilingual Hindi-English LLM specialized in Indic language understanding. We also evaluate Krutrim-2-Instruct [56], a Mistral-NeMo (Mistral AI and NVIDIA)–based model trained on diverse domains, including Indic languages, and fine-tuned with direct preference optimization to improve alignment and reasoning for Indian contexts. For proprietary baselines, we include GPT-5 [30], GPT-4o [31], and Claude-3.5-Sonnet [57], which serve as state-of-the-art performance references despite their closed parameter counts.

For open-weight multilingual models, we include a range spanning smaller (7B-9B) to larger (27B-70B) architectures. Alongside these, we also include Sarvam-M (Sarvam AI) [58], a state-of-the-art open-source hybrid Indic LLM built on Mistral-Small, designed to enhance reasoning in Indian languages through supervised fine-tuning and reward-based reinforcement learning. Since Indic LLMs are still emerging, our evaluation also includes small to mid-sized versions currently available (7B-12B). This selection allows for a balanced comparison across Indic, multilingual, and proprietary systems, highlighting the relative strengths and limitations of each category.

With LLMs, a precise and well-structured prompt is paramount for shaping responses and ensuring that the model focuses on the most relevant aspects of the input. Recent research [59] has shown that even minor lexical variation—sometimes just a single-word difference—in prompts can significantly impact model performance on downstream tasks. To accurately determine topic and subtopic levels in our task, we experimented with multiple prompt variations on a small, representative subset of our dataset. This iterative process allowed us to refine the prompts efficiently before running the large-scale evaluation. Smaller open-weight models were evaluated locally on a graphics processing unit with 48GB of memory (eg, NVIDIA RTX A6000), while proprietary models were accessed via application programming interface–based inference. The final prompt template used across all models is shown in Table S1 in Multimedia Appendix 2. The prompt template also requested a self-reported confidence score (0.0‐1.0); however, this value was extracted for future analysis but was not used in this evaluation.

### Qualitative Error Analysis

To better understand the strengths and limitations of the models, we conducted a qualitative error analysis of the 2 best-performing models in each family (proprietary, multilingual open-weight, and Indic models). For each model, we sampled 50 misclassified test queries stratified by topic and subtopic. The same 2 annotators who created the dataset served as reviewers and independently coded thematic errors; disagreements were resolved by consensus. Results are summarized in the “Error Analysis” section, with examples in Table 4.

**Table 4.** Performance of 6 models on representative sexual and reproductive health queries (SRHQ) in the SRHQ-India dataset queries at the topic-subtopic level.

Error types	Query (Hinglish→English)	Ground truth (Topic→Subtopic)	GPT-5	Claude-3.5-Sonnet	Llama-3.3-70B-Instruct	Gemma-3-27B-IT	Sarvam-M	Llama-3-Gaja-Hindi-8B
C1	Safaiya kaise karwate hain? (How is an abortion done?)	Pregnancy and ^a^PNC→Abortion	✓^b^	✓	—^c^	—	—	—
C2	Pregnancy me 5 month me vomiting hoti h to kya kre (What should I do if I am vomiting in the 5th month of pregnancy?)	Pregnancy and PNC→Antepartum	✓	✓	—	—	✓	—
C3	Family planning Muslim community me accept hain kya? (Is family planning accepted in the Muslim community?)	Contraception and Family Planning→Family Planning Queries	—	—	✓	✓	—	✓
C3	Masik pali aane se Bhagvan ke pas kau nahi jana chahiye? (Why should one avoid going to god/temples during periods?)	Other→Cultural, Religious, or Moral Norms	✓	✓	—	—	✓	—
C4	1 sal se bacha rukhne ke liye try kar rahe hai lekin nahi rukh raha hai to kya karna padega? (We have been trying to have a child for one year, but it has not happened. What should we do?)	Pregnancy and PNC→Infertility	✓	✓	—	—	—	—
C4	Family planning may agar koi mahila test tube karvati hai to kya hota hai? (In family planning, if a woman undergoes test-tube treatment, what happens?)	Pregnancy and PNC→Infertility	—	—	—	—	—	—
C5	Kitni der jinda rahte hain sperm? (How long do sperm stay alive?)	Sexual and Vaginal Health→Reproductive Anatomy	—	✓	—	—	—	—

a PNC: postnatal care.

b ✓ indicates correct topic-subtopic classification.

c — indicates incorrect topic-subtopic classification

### Ethical Considerations

We used deidentified secondary data shared by the Myna Mahila Foundation. Since the dataset was fully deidentified before being shared, no personally identifiable information was accessible to the research team at Emory University. This study did not involve direct interaction with human participants, and no additional data collection was conducted. Approval for the use of this secondary dataset was obtained from Emory University’s Institutional Review Board (Protocol #2025P011010). Data security measures were implemented to maintain confidentiality, and the dataset was stored on a secure, password-protected system. Only authorized personnel had access to the data. As the study relies on pre-existing deidentified data, the risk to individuals is minimal.

## Results

### Overview

**Figure 2.**
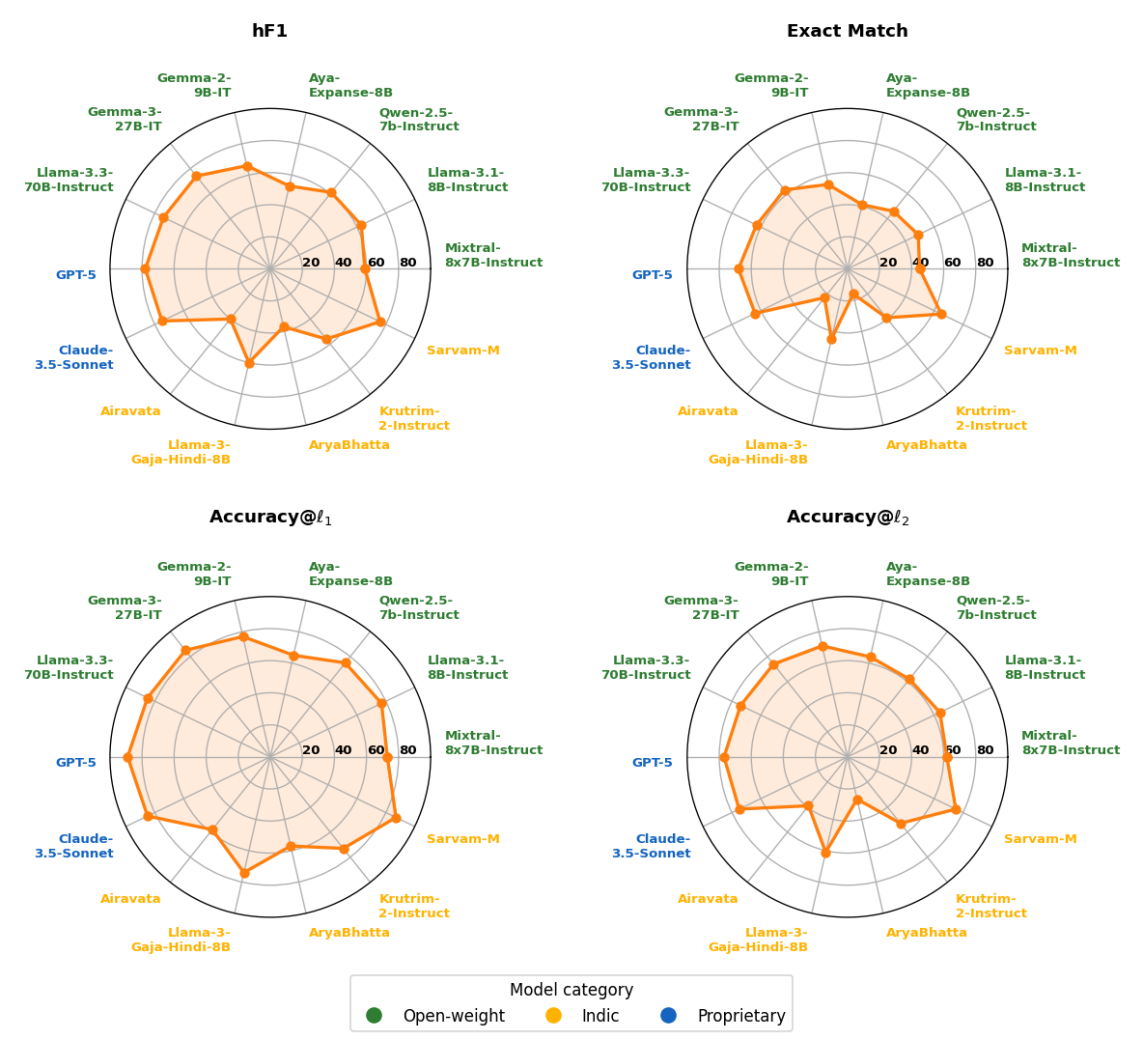
Visual comparison of model performance on the sexual and reproductive health queries (SRHQ) dataset across 4 evaluation metrics: hierarchical *F*_1_(h*F*_1_), Exact Match, Accuracy@l_1_, and Accuracy@l_2_. The figure highlights relative strengths and differences among open-weight, Indic, and proprietary models.

We now present the results of the hierarchical classification of SRHQ with LLMs. Table 5 compares model performance on the hierarchical classification across 3 types of models: open-weight, Indic (LLMs fine-tuned for Indian languages), and proprietary. Figure 2 provides a visual comparison across 4 evaluation metrics: hierarchical *F*_1_(h*F*_1_), Exact Match, and accuracy at the topic (l_1_) and subtopic (l_2_) levels. Although we also evaluated GPT-4o, its performance was less than 1.5% lower than GPT-5 across metrics. Bootstrap-based 95% CIs further show substantial overlap between GPT-5 (h*F*_1_=0.784, 95% CI 0.774‐0.795) and GPT-4o (h*F*_1_=0.779, 95% CI 0.768‐0.789), indicating that performance differences between the 2 proprietary systems are not statistically distinguishable (Table S2 in Multimedia Appendix 2). For consistency and balanced cross-category comparisons, we focus here on GPT-5 and Claude-3.5-Sonnet as representative proprietary models.

**Table 5.** Performance comparison of different models in a zero-shot setting on the sexual and reproductive health queries (SRHQ) dataset. Open-weight, proprietary, and Indic models are distinctly highlighted for clarity. All Indic models are open weights. In each category, the best-performing model is highlighted in **bold**, and the second-best is underlined.

Category and model	#Params	h*F*_1_ (hierarchical *F*1)	Exact Match	Accuracy@l_1_	Accuracy@l_2_
Open-weight models					
Mixtral-8 × 7B-Instruct	7B	0.593	0.453	0.733	0.617
Llama-3.1-8B-Instruct	8B	0.630	0.491	0.769	0.638
Qwen-2.5-7b-Instruct	7B	0.605	0.463	0.747	0.619
Aya-Expanse-8B	8B	0.528	0.411	0.646	0.636
Gemma-2-9B-IT	9B	0.657	0.544	0.770	0.706
Gemma-3-27B-IT	27B	0.739	0.629	0.849	0.741
Llama-3.3-70B-Instruct	70B	**0.742**	**0.630**	**0.853**	**0.738**
Indic models					
Airavata	7B	0.404	0.226	0.581	0.389
Llama-3-Gaja-Hindi-8B	8B	0.596	0.452	0.740	0.610
AryaBhatta	8.5B	0.365	0.157	0.574	0.273
Krutrim-2-Instruct	12B	0.558	0.386	0.731	0.527
Sarvam-M	24B	**0.757**	**0.647**	**0.867**	**0.747**
Proprietary models					
GPT-5	—^a^	**0.784**	**0.683**	**0.886**	**0.771**
GPT-4o^b^	—	0.779	0.675	0.882	0.764
Claude-3.5-Sonnet	—	0.745	0.639	0.851	0.751

a Not available.

b Although GPT-4o was the second-best-performing proprietary model, its performance differed from GPT-5 by less than 1.5% across metrics. Therefore, GPT-5 and Claude-3.5-Sonnet were selected as representative proprietary models for balanced cross-category comparisons in the main analysis.

Proprietary systems delivered the highest and most consistent performance, with GPT-5 achieving the best results across all metrics, with h*F*_1_=0.784, Exact Match=0.683, and accuracies of 0.886 at l_1_ and 0.771 at l_2_. Claude-3.5-Sonnet closely followed (h*F*_1_=0.745 and Exact Match=0.639), exceeding 0.85 topic-level accuracy and 0.75 subtopic-level accuracy.

Among open-weight models, the strongest performers were Llama-3.3-70B-Instruct and Gemma-3-27B-IT, which achieved performance close to the proprietary models, with h*F*_1_ scores of 0.742 and 0.739 and Exact Match scores of 0.630 and 0.629, respectively. Both models exceeded 0.84 in topic-level accuracy (l_1_) and maintained strong subtopic accuracy (l_2_ ≈ 0.74) with scores of 0.738 and 0.741. Mid-sized models such as Gemma-2-9B-IT also performed competitively (h*F*_1_=0.657, Exact Match=0.544, l_1_=0.770, and l_2_=0.706), whereas smaller models such as Aya-Expanse-8B underperformed across metrics (h*F*_1_=0.528, Exact Match=0.411) and struggled particularly at the subtopic level (l_2_=0.636). Figure 2 illustrates this scaling effect clearly, with larger multilingual models covering a broader area across h*F*_1_, Exact Match, and both accuracy levels compared to their smaller counterparts.

Indic models showed the widest variability, with Sarvam-M emerging as a strong outlier, achieving h*F*_1_=0.757 and Exact Match=0.647. Compared to GPT-5, Sarvam-M fell short by only 3.4% in h*F*_1_. At the topic level, it reached an accuracy of l_1_=0.867, and at the subtopic level l_2_=0.747, only slightly below the top-performing model GPT-5 (0.886 and 0.771, respectively). This indicates that Sarvam-M is the strongest free and open-weight alternative to proprietary models, considering the restricted access and cost associated with proprietary models. Other Indic models performed substantially lower; the second-highest-performing Indic model was Llama-3-Gaja-Hindi-8B, achieving an h*F*_1_ of 0.596, an Exact Match of 0.452, and accuracies of 0.740 at l_1_ and 0.610 at l_2_. Interestingly, it outperformed the larger Krutrim-2-Instruct (12B; OLA Krutrim Team) (h*F*_1_=0.558, Exact Match=0.386, l_1_=0.731, and l_2_=0.527), while Airavata demonstrated lower performance across metrics (h*F*_1_=0.404, Exact Match=0.226, l_1_=0.581, and l_2_=0.389). These comparisons indicate that parameter count alone does not guarantee superior performance; factors such as model design and training data quality appear to play a more decisive role. By contrast, Airavata and AryaBhatta consistently trailed across all metrics, clustering at the lowest end of performance in both topic- and subtopic-level accuracy.

The results highlight a consistent performance hierarchy across model categories. Proprietary systems dominated overall, while large open-weight multilingual models narrowed the gap across all metrics, and Indic models demonstrated both promise for local contexts and limitations. Sarvam-M stood out as a competitive alternative, while other Indic models struggled to identify user intent in code-mixed settings. The combination of Table 5 and Figure 2 underscores not only differences in overall performance but also the critical difficulty of achieving accurate subtopic-level predictions in mixed-language SRH queries.

**Figure 3.**
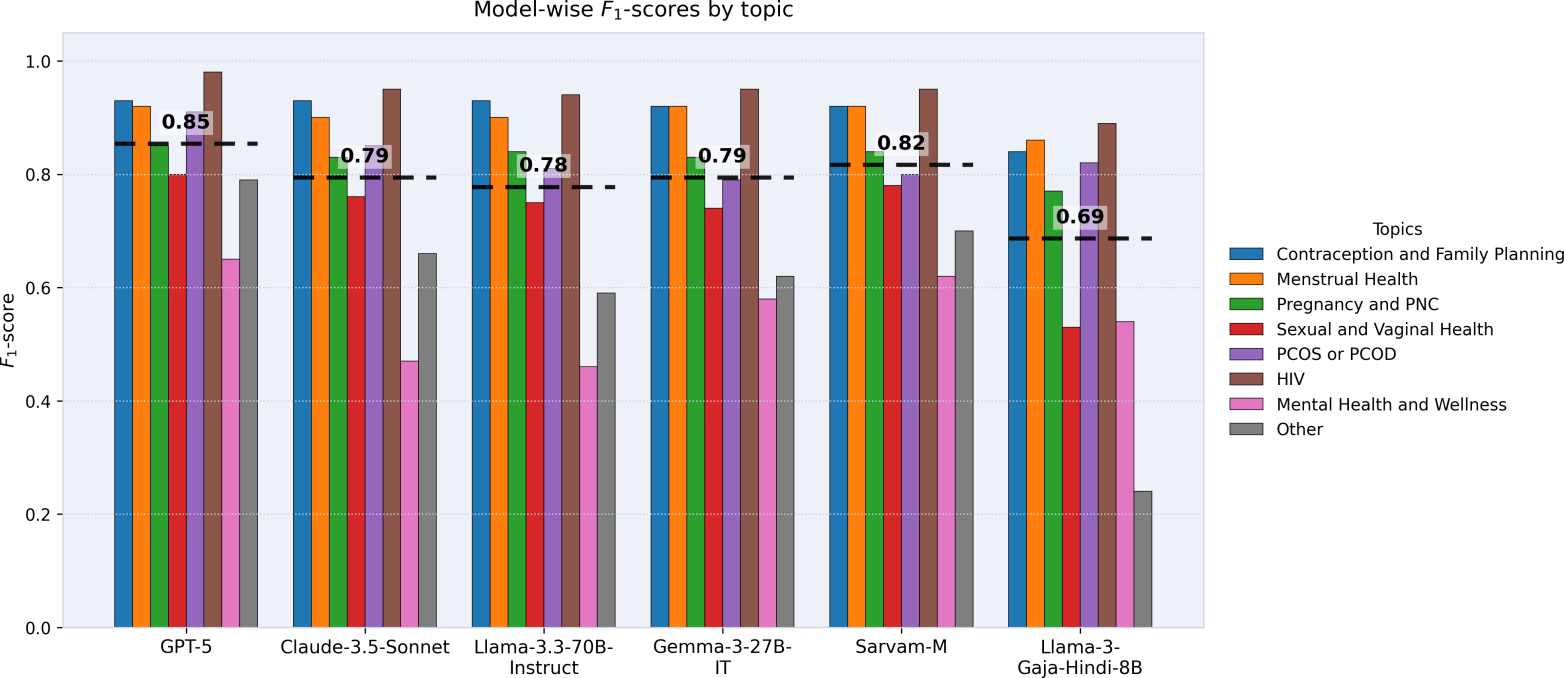
Model-wise topic-level *F*_1_ scores across the sexual and reproductive health queries (SRHQ)-India dataset. Each bar represents the flat *F*_1_ score for a specific topic, while the dotted lines indicate the macro-average of topic-level *F*_1_ scores across all topics for each model. Results highlight differences between proprietary, open-weight, and Indic models. HIV: human immunodeficiency virus; PCOD: polycystic ovarian disease; PCOS: polycystic ovary syndrome; PNC: postnatal care.

To further examine performance differences, Figures3  4 present *F*_1_ scores across SRHQ-India topics. Figure 3 highlights per-topic *F*_1_ scores alongside model-wise averages, where the reported average *F*_1_ is the macro-average of flat, topic-level *F*_1_ scores computed across all topics for each model. Proprietary model GPT-5 achieved the highest overall average *F*_1_ score (0.85), while Sarvam-M emerged as the strongest open-weight Indic alternative with an average of 0.82, with performance comparable to Claude-3.5-Sonnet (0.79) and other open-weight models such as Llama-3.3-70B (0.78) and Gemma-3-27B (0.79). Other Indic models, such as Llama-3-Gaja-Hindi-8B (CognitiveLab), showed weaker averages and wider variability, particularly in nuanced domains.

**Figure 4.**
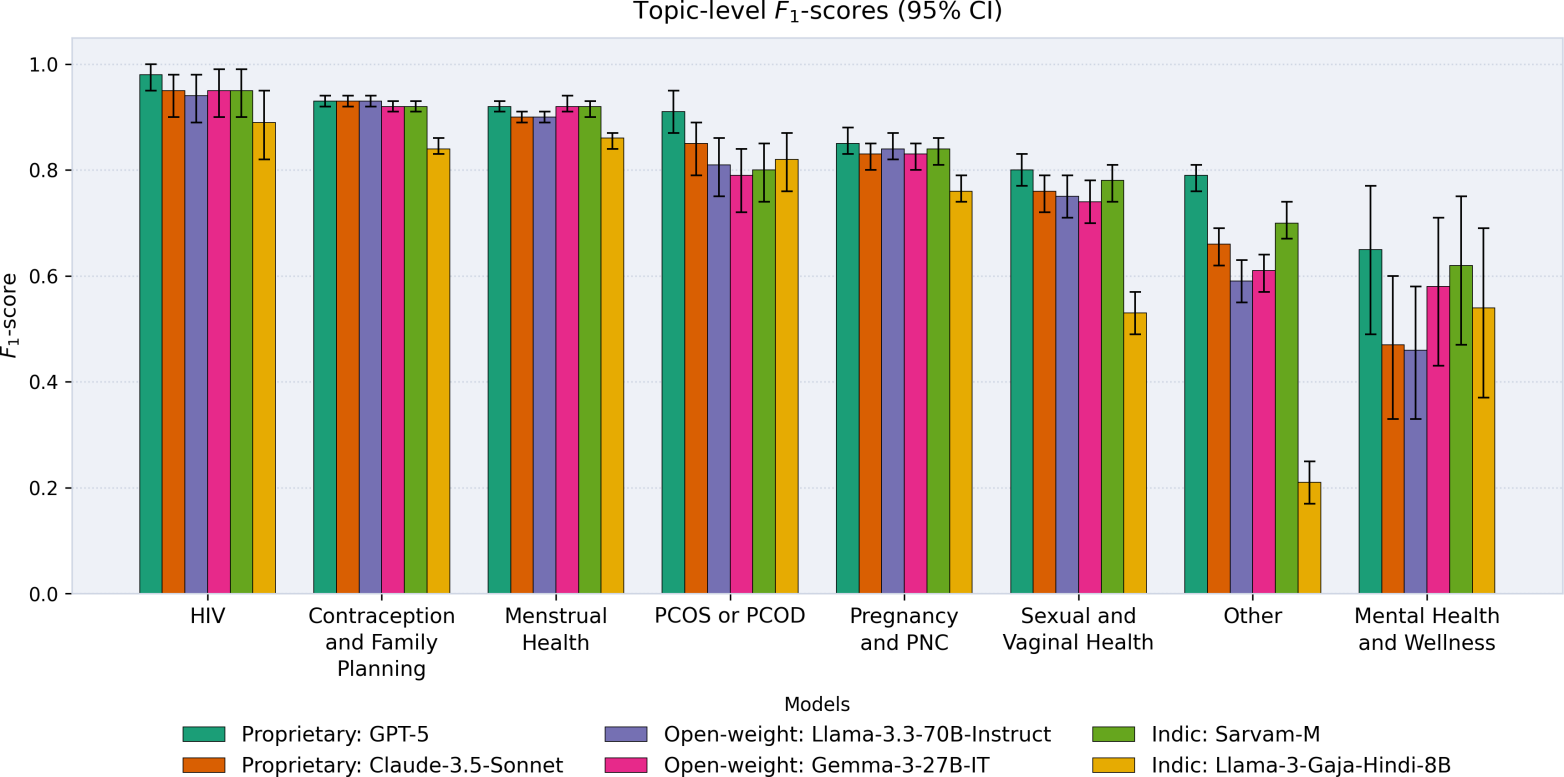
Topic-level *F*_1_ scores with 95% CIs across sexual and reproductive health queries (SRHQ)-India. Each bar represents the *F*_1_ score for a specific topic, with error bars indicating 95% CIs estimated via bootstrap resampling. Results highlight performance differences across proprietary, open-weight, and Indic models. HIV: human immunodeficiency virus; PCOD: polycystic ovarian disease; PCOS: polycystic ovary syndrome; PNC: postnatal care.

Figure 4 provides a closer look at topic-level differences across models. Proprietary systems maintained stability across nearly all categories, performing strongest in human immunodeficiency virus, Contraception and Family Planning, and Menstrual Health, which require precise and time-sensitive classification. Although human immunodeficiency virus was represented in fewer queries, performance remained consistent across models, suggesting less variability in how these queries are expressed. Sarvam-M again demonstrated competitive results, closely tracking proprietary and large multilingual models in categories such as Contraception and Family Planning, Menstrual Health, and polycystic ovary syndrome or polycystic ovarian disease. However, all models showed relative declines in Sexual and Vaginal Health, and Mental Health and Wellness, where queries frequently overlap with adjacent categories and require additional context for a clear-label decision. In these topics, diverse expressions of intent and limited sample sizes contribute to greater uncertainty, particularly for Mental Health and Wellness, where even a small number of misclassifications can substantially affect performance estimates. These findings highlight that while proprietary systems remain strongest, large multilingual open-weight models substantially close the gap, and Sarvam-M stands out as a promising Indic alternative. Yet, across all families, accurate subtopic-level predictions in code-mixed SRH queries continue to be the most persistent challenge.

**Figure 5.**
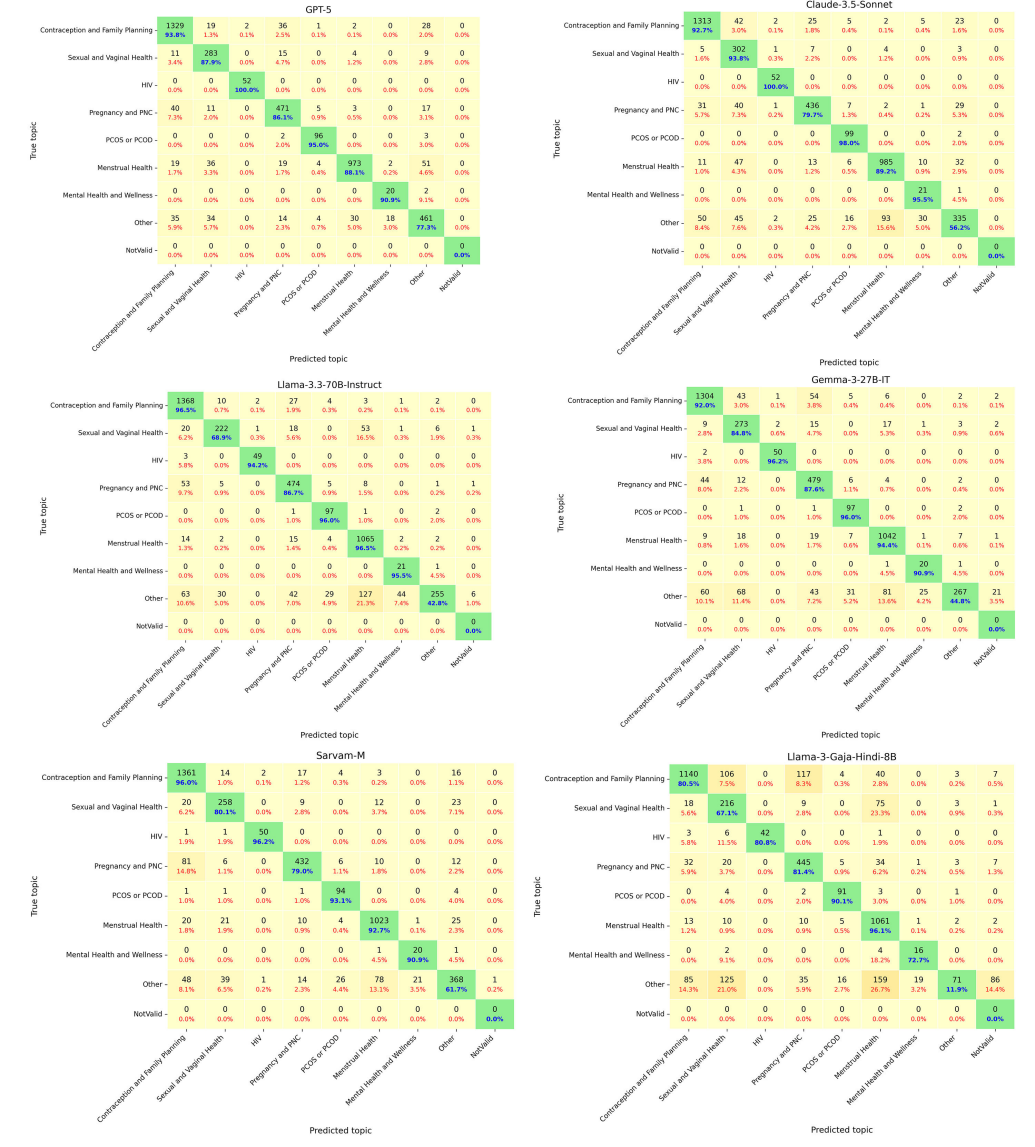
Topic-level confusion matrices for 6 models representing proprietary, open-weight, and Indic categories. Each cell shows counts (black) and row-normalized percentages. Correct classifications appear in green diagonal cells with percentages in blue. Misclassifications appear in off-diagonal yellow cells, with percentages shown in red. “NotValid” denotes predictions that do not belong to any valid topic. HIV: human immunodeficiency virus; PCOD: polycystic ovarian disease; PCOS: polycystic ovary syndrome; PNC: postnatal care.

To further examine where models succeed and fail, Figure 5 presents confusion matrices for proprietary, open-weight, and Indic models, highlighting recurring misclassification patterns. Consistent weaknesses appeared in overlap-prone and context-dependent topics such as Other, Mental Health and Wellness, and Sexual and Vaginal Health, where misclassifications were frequent and often collapsed into broader categories. Proprietary systems, particularly GPT-5, demonstrated the most balanced performance across all topics, while Claude-3.5-Sonnet showed sharper drops in Other categories despite strong results elsewhere. Open-weight models (Gemma-3-27B and Llama-3.3-70B) performed competitively but exhibited greater instability in culturally sensitive areas, including frequent misclassification of Sexual and Vaginal Health queries into the Pregnancy and PNC or Other category. Among Indic models, Sarvam-M emerged as the strongest, closely mirroring proprietary models’ performance by correctly classifying over 90% of queries in Menstrual Health, Contraception and Family Planning, and Mental Health and Wellness. However, it still struggled in fine-grained intent recognition, with frequent misclassification of Contraception and Family Planning into the Pregnancy and PNC or Menstrual Health category. By contrast, smaller Indic systems such as Llama-3-Gaja-Hindi-8B displayed widespread confusions across sensitive categories and a notable tendency to produce “NotValid” predictions, where models generated labels not included in the predefined SRH hierarchy shown in the prompt. For clarity in Figure 5, predictions that fall outside the predefined SRH hierarchy are grouped under a “NotValid” category*.* These out-of-hierarchy outputs receive no credit in the hierarchical evaluation. These cases underscore the limited ability of smaller Indic models to consistently map inputs to the defined label structure and reflect the challenges related to limited training data and weaker alignment with the task structure.

### Error Analysis

To better understand model behavior beyond aggregate metrics, we conducted a qualitative analysis of errors from 6 representative models (GPT-5, Claude-3.5-Sonnet, Llama-3.3-70B-Instruct, Gemma-3-27B-IT, Sarvam-M, and Llama-3-Gaja-Hindi-8B) spanning proprietary, multilingual open-weight, and Indic categories. For each model, we sampled 50 misclassified queries and performed a detailed review of their errors. Table 4 presents examples of misclassification. Additionally, we included error analysis for GPT-5 and GPT-4o in Table S3 in Multimedia Appendix 2, highlighting cases where GPT-4o misclassified queries that GPT-5 handled correctly.

#### C1: Lexical Ambiguity and Euphemisms

In Table 4, row 1, the query *“*safaiya kaise karwate hain?” translates to “How is an abortion done?” and should be classified as Pregnancy and PNC→Abortion. However, only the proprietary models assigned the topic and subtopic correctly; both open-weight and Indic models misclassified the query under Sexual and Vaginal Health, Menstrual Health, or General Health Queries*.* As detailed in Table S4 in Multimedia Appendix 3, model-generated explanations indicate that these errors stem from the literal interpretation of the colloquial term “safaiya” as hygiene, sanitary products, or general cleaning practices. In practice, however, the term is commonly used to refer to the abortion evacuation procedure in the Indian context [60]. This example highlights how culturally embedded euphemisms widely used in Indian contexts can mislead models if culturally specific meanings are not recognized.

#### C2: Misclassifying Stage in the Reproductive Health Journey

As shown in Table 4, row 2, models frequently confused different pregnancy stages. The query *“*Pregnancy me 5 month me vomiting hoti hya to kya kre” translates to “What should I do if I am vomiting in the 5th month of pregnancy?” which pertains to the antepartum stage (pregnancy period). Of the 6 best-performing models, only GPT-5, Claude-3.5-Sonnet, and Sarvam-M predicted this correctly. The remaining models classified this query under more general Pregnancy Information, thereby failing to distinguish the specific stage of the reproductive health journey.

As illustrated in Table S5 in Multimedia Appendix 3, these models recognized the pregnancy context but did not incorporate gestational timing into their subtopic selection. This distinction is clinically important, as symptoms such as vomiting have different implications and recommended guidance during the antepartum vs postpartum periods. Assigning such queries in overly broad subcategories such as *“*Pregnancy Information” dilutes the specificity of the advice that models are expected to provide and risks misalignment with clinical best practices.

#### C3: Cultural and Religious Context Error

In Table 4, row 3, the user query “Family planning Muslim community me accept hain kya?” translates to “Is family planning accepted in the Muslim community?” reflects a user’s religious concern regarding the acceptability of family planning. In our evaluation, both open-weight models correctly assigned the topic and subtopic, while others—including proprietary and Indic models—placed the query under Cultural, Religious, or Moral Norms. Model-generated reasons (Table S6 in Multimedia Appendix 3) indicate that these models focused on the religious framing of the questions rather than their underlying health-related intent. Similarly, in row 4, the query “Masik pali aane se Bhagvan ke pas kyo nahi jana chahiye?” (Why should one avoid going to temples during menstruation?) reflects a culturally and religiously grounded belief rather than a medical concern. GPT-5, Claude-3.5-Sonnet, and Sarvam-M correctly classified this query under Cultural, Religious, or Moral Norms. In contrast, 3 other models, including both open-weight and Indic models, classified it under health-related categories such as Menstrual Health or Misconceptions and Myths. Model-generated reasons (Table S6 in Multimedia Appendix 3) suggest that these systems focused on biological or psychological interpretations of menstruation rather than the user’s culturally grounded concern.

In both examples above, the alternative categorizations are semantically plausible given the cultural framing of the user queries; therefore, we treat these cases as a routing-level issue rather than a linguistic error. We later discuss how we might deal with such ambiguous cases when classifying user intent in the “Limitations and Future Works” subheading.

#### C4: Misunderstanding Reproductive Intent

This category captures cases where models failed to recognize the user’s reproductive intent, often reversing it from wanting to conceive to wanting to avoid conception. For example, in Table 4, row 5, the query “1 sal se bacha rukhne ke liye try kar rahe hai lekin nahi rukh raha hai to kya karna padega?” translates to “We have been trying to have a child for one year, but it has not happened. What should we do?“ and reflects the issue of infertility, a deeply personal and emotionally sensitive topic. However, only the proprietary model classified this query correctly. Model-generated reasons from other systems (Table S7 in Multimedia Appendix 3) indicate that the colloquial phrase “bacha rukna,” which in India commonly means conceiving, was interpreted literally as preventing pregnancy. As a result, these models prioritized Contraception and Family Planning subtopics such as Family Planning Queries and Contraceptive Effectiveness and Duration*.* While such interpretations are not inherently logical given the taxonomy, they diverge from the user’s underlying intent of seeking fertility-related guidance.

A similar misalignment occurred in row 6 with the query *“*Family planning me agar koi mahila test tube karvati hai to kya hota hai?” (“In family planning, if a woman undergoes a test tube procedure, what happens?”). Here, the user was referring to IVF, a fertility treatment aimed at achieving pregnancy. Yet all models categorized the query under Family Planning or Sterilization rather than Infertility. This demonstrates how culturally specific terms such as “test tube karvati hai” *can in*vert user intent, classifying conception-seeking queries as if they were about contraception.

From an SRH perspective, such errors are critical, since infertility queries require guidance on fertility assessment and treatment. Misclassifying these queries risks invalidating user concerns, reinforcing stigma, as infertility in India is often associated with blame and silence, particularly for women [61]. Treating these queries as matters of pregnancy avoidance could further marginalize users seeking support.

#### C5: Misclassifying Questions About Bodily Anatomy or Processes

In Table 4, row 7, the query *“*Kitni der jinda rahte hain sperm?” translates to “How long do sperm stay alive?“ and should be categorized under Sexual and Vaginal Health, subtopic Reproductive Anatomy. Only 1 model (Claude-3.5-Sonnet) correctly identified both the topic and subtopic. Other models routed the query to categories such as Sexual and Vaginal Health, Contraception and Family Planning, Pregnancy and PNC, or General Health Queries (Table S8 in Multimedia Appendix 3). Model-generated reasons indicate that many systems overassociate the term sperm with sexual behavior, contraception, or pregnancy risk rather than recognizing it as a biological question. Such routing decisions are consequential, as users seeking factual information about sperm viability may instead receive guidance about sexual behavior or contraception, reducing clarity and potentially misdirecting them away from accurate, evidence-based biological information.

## Discussion

### Principal Findings

Our evaluation shows that both model family and training data alignment play an important role in hierarchical SRH intent classification, alongside model scale. Proprietary systems still lead, but the gap narrows when models are trained on culturally aligned and domain-relevant data. Sarvam-M, an Indic-first model, emerged as the strongest Indic system and achieved performance comparable to top-performing proprietary models and the best large open-weight baselines such as Llama-3.3-70B-Instruct and Gemma-3-27B-IT. It is also important to consider that Sarvam-M is larger than the other Indic models evaluated, and its performance likely reflects a combination of increased model capacity and Indic-focused training data. While these results do not fully disentangle the effects of scale and data quality, they suggest that culturally aligned resources, rather than parameter count, are the primary drivers of performance on code-mixed intent classification.

Across the 6 best-performing models, we observed a consistent pattern of subtopic misclassification. In Pregnancy and PNC*,* antepartum and postpartum concerns are frequently conflated, and abortion-related queries are sometimes assigned to the “Pregnancy Information” subcategory. In “Sexual and Vaginal Health,” euphemisms such as *“*safaiya or safai*”* (colloquial for abortion) were misinterpreted literally as “cleaning.” Similarly, culturally embedded phrases such as “bacha rukna” (conceiving) are sometimes misinterpreted as preventing pregnancy, and “test tube karvati hai” (referring to IVF) is sometimes treated as sterilization or contraception [62]. This reflects how health communication in rural, low-literacy settings often relies on indirect, gendered, and culturally mediated channels [63]. Accounting for these cultural expressions is essential to improve classification accuracy and ensure that clinically relevant questions are not overlooked. We also observed that SRH queries could be misrouted as Cultural, Religious, or Moral Norms, even when the underlying intent is to seek health guidance or clinical information. A user query could also plausibly fit into multiple other categories, as we observed. Future work may explore culturally informed prompt design and lightweight in-context examples that expose models to common SRH euphemisms and colloquial expressions. Additionally, to address overlapping topics, researchers could explore alternative approaches that account for such ambiguity, such as ranked classification [64].

In the *“*Other” category, even strong models such as Llama-3-Gaja-Hindi-8B and Gemma-3-27B-IT frequently assigned queries to new topics outside the defined hierarchy: we consider that kind of misclassification as “Not Valid” predictions (Figure 5). These cases arise when models generate unintended or unsupported labels that are not part of the hierarchical classification structure, reflecting models’ tendency toward hallucinations [65] or schema misalignment [66]. Our hierarchical framework requires a correct topic prediction before the subtopic evaluation. Accordingly, “NotValid*”* predictions are treated as complete classification failures and receive no credit in the hierarchical *F*_1_ score. Overall, these patterns indicate that models struggle to reliably map culturally and contextually grounded queries to the specified hierarchy. This limitation is essentially critical in SRH contexts, where errors at the topic level prevent meaningful subtopic interpretation and risk misrouting sensitive queries. High precision at the fine-grained intent level is paramount to ensure safe guidance and avoid reinforcing stigma.

When compared to prior chatbot efforts, our findings highlight the importance of a hierarchical classification approach. SnehAI [15], an SRH-focused conversational agent deployed in India, demonstrated feasibility but relied on rule-based methods, which restrict its ability to process open-ended, code-mixed, or culturally nuanced queries. Health-Pariksha [38], while not designed specifically for SRH, evaluated several LLMs on real-world health care queries, measuring the factual correctness, semantic similarity, coherence, and conciseness of the model responses. Although this approach contributed to improving trustworthiness, it did not address the challenges of intent recognition or hierarchical classification. In contrast, our framework directly targets the layered structure of SRH queries, enabling distinction between broad categories and their fine-grained subcategories. This supports classifications that are both clinically appropriate and culturally relevant. Through our evaluation, proprietary models emerged as the most reliable for hierarchical classification, but notably, Sarvam-M emerged as the second-highest performing model overall—outscoring Claude-3.5-Sonnet and closely matching Llama-3.3-70B-Instruct. Given that Sarvam-M is open weight and free to use, it represents a promising alternative to proprietary systems, showing that with robust data and cultural adaptation, Indic models can deliver competitive performance.

### Limitations and Future Works

This study has several limitations. First, we focused on Hinglish as an illustrative case of code-mixed SRH queries, while many other languages and code-mixing patterns are used in India. Second, the hierarchical intent schema was developed for our setting and user population; researchers working in other regions should adapt label definitions to their context and target users. Third, although we evaluated many more models, several were excluded from the final set presented in this paper, either due to poor performance on code-mixed data or the cost of highly parameterized models. Fourth, our dataset is limited in size; certain topics and subtopics are underrepresented, resulting in lower statistical power for these categories, and performance estimates for these topics should be interpreted as preliminary. Fifth, our evaluation was conducted in a strict zero-shot setting, where models were not provided with the detailed subtopic definitions used by human annotators. This design allows us to assess how models infer intent from real-world, code-mixed queries without semantic scaffolding. Finally, our evaluation treats intent as a single topic, even though some queries plausibly map to more than 1 topic, motivating the exploration of alternative modeling approaches that can better capture such ambiguity.

In the future, we plan to extend our work to support additional Indian languages and code-mixing patterns, enriching both the breadth of topics and the depth of subtopic coverage in collaboration with the Myna Mahila Foundation. We also plan to evaluate in-context prompting strategies and to explore ranked classification [67] and hierarchical selective classification [64] approaches, which allow models to better account for ambiguity in hierarchical intent prediction.

### Implications

This study highlights the importance of hierarchical classification for SRH applications in low-resource settings. By capturing intent at both the topic and subtopic levels, this framework improves contextual precision, supports safer dialogue systems, and enables public health organizations to design targeted interventions. At the same time, our findings show that LLMs often struggle with euphemisms and sociocultural framing, highlighting the need for systems that are socially attuned as well as technically accurate. Our evaluation offers a starting benchmark that can guide the development of culturally aligned, open-source LLM tools for low-resource health care contexts. More broadly, this work contributes to the discourse on health equity in AI by addressing the linguistic and cultural barriers that limit access to reliable health information for marginalized populations.

### Conclusions

In this study, we evaluated the zero-shot performance of proprietary, open-weight multilingual, and instruction-tuned Indic LLMs on the hierarchical classification of SRH queries. Our findings reveal that the proprietary model (GPT-5) was the most reliable, while Sarvam-M emerged as the strongest Indic model and performed competitively with Claude-3.5-Sonnet and large open-weight models such as Llama-3.3-70B-Instruct. This highlights the importance of culturally aligned and domain-relevant training data alongside model scale. Our error analysis further revealed that models frequently misclassified queries that involved euphemisms, cultural or religious language, or time-sensitive concerns, underscoring the importance of capturing nuance at both topic and subtopic levels. Misclassifications in these areas risk unsafe guidance and reinforcing stigma, particularly around sensitive issues such as abortion and infertility. By introducing a benchmark evaluation framework, this study supports the development of open-source multilingual models for SRH and advances culturally aligned, socially responsive AI systems to better serve the health information needs of underserved communities.

The source code used in this study, including scripts for model evaluation, hierarchical metric calculation (h*F*_1_), bootstrap resampling, and CMI calculation, is publicly available at a GitHub repository [68] to support future research.

## Supplementary material

10.2196/86545 Multimedia Appendix 1 Annotation guidelines for hierarchical topic-subtopic labeling of the sexual and reproductive health queries (SRHQ-India dataset).

10.2196/86545 Multimedia Appendix 2 Prompt template and statistical performance analysis of zero-shot hierarchical classification performance across open-weight, Indic, and proprietary large language models (LLMs), including a comparative analysis of GPT-5 and GPT-4o on representative user queries.

10.2196/86545 Multimedia Appendix 3 Model-generated reasons for predicted hierarchical classifications across representative sexual and reproductive health (SRH) queries, reported for all evaluated models.

## Abbreviations

AI: artificial intelligence
CMI: Code-Mixing Index
IVF: in-vitro fertilization
LLM: large language model
PNC: postnatal care
SRH: sexual and reproductive health
SRHQ: sexual and reproductive health queries
