# Supplementary material for: Understanding User Intent in Code-Mixed Sexual and Reproductive Health Queries in Urban India: Hierarchical Classification Approach Using Large Language Models
Source: J Med Internet Res. 2026 Mar 24;28:e86545. doi: 10.2196/86545 (PMC13012004; doi:10.2196/86545)
Supplement: Multimedia Appendix 1 [file jmir-v28-e86545-s001.docx]

Annotation Guidelines

Objective: Annotation for user data on Sexual and Reproductive Health (SRH), classified into 8 topics and 40 subtopics for in-depth understanding of the user queries related to SRH. Our aim is to evaluate large language models, taking a hierarchical classification approach to identify user intent in SRH queries.

General Instructions:

1. Each user query must be annotated with:

- One primary topic (from the 8 main topics mentioned below)
- One subtopic based on the description mentioned under the subtopic.

1. Understand the user’s concern in full, if confused highlight for review.
2. Choose the most relevant main topic and do not infer the query. Use the description provided under each subtopic to categorise the queries in case of need for clarity.
3. Choose the most relevant subtopic under the selected topic.
4. Only annotate based on explicit or clearly implied content mentioned in the query.
5. For subtopics such as “*Diet and Nutrition*”, any questions specifically related to areas like diet during pregnancy or diet for PCOS will be categorized under those specific subtopics rather than the *“Diet and Nutrition”* subtopic.
6. Use “General Health” under the “*Other”* topic when the query is health-related but not related to SRH and does not fit well under any predefined topic.
7. If anything is unclear Highlight or mark for review if the content is ambiguous or unrelated to health.

EXAMPLES:

1. Periods agar monthly na aaye to kya karen?

*(Translation: What should I do if my periods are not regular every month?)*

Topic: Menstrual Health

Subtopic: Menstrual Cycle Information

1. Gabhnirodhak goliyan lene se pehle mujhe doctor se kya puchna chahiye? *(Translation: What should I ask the doctor before taking birth control pills?)*

Topic: Contraception and Family Planning

Subtopic: Usage Guidance

NOTE:

1. Ensure all data is anonymized before annotation.
2. Do not attempt to re-identify individuals.
3. Report any data that appears to contain identifiable information.
4. Regular meetings for annotation consistency checks and clarifications

******* *Each section is organized by a* ***Topic****, followed by its corresponding* ***subtopics*** *listed below.*

## **Topic: Pregnancy and PNC:**

1. **Abortion**

The termination of a pregnancy by removal or expulsion of an embryo or fetus. The unmodified word of abortion generally refers to induced abortion, or deliberate actions to end a pregnancy.

- Medical and surgical methods of abortion.
- Legal and ethical considerations of abortion in specific contexts (e.g., India’s abortion laws).
- Post-abortion care and recovery.
- Emotional and psychological support after an abortion.

1. **Miscarriage**

A miscarriage, also known as early pregnancy loss, is the spontaneous loss of a pregnancy before the 20th week

- Causes, symptoms, and management options.
- Vaginal bleeding and passing of clots
- Loss of pregnancy symptoms
- Psychological impact and coping strategies.

1. **Antepartum**

The stage from contraception to before childbirth

- Prenatal care essentials (checkups, tests, ultrasounds).
- Managing pregnancy symptoms (nausea, fatigue, etc.).
- Nutrition and exercise

1. **Postpartum:** After childbirth

- Physical recovery after childbirth.
- Emotional health (postpartum depression/anxiety).
- Newborn care and contraception.

1. **Infertility**

- Assisted with reproductive technology.
- Overview of methods (e.g., IVF, ICSI, and surrogacy).
- Success rates and influencing factors.
- Causes, diagnosis, and treatment options.
- Emotional challenges and support resources.

1. **Pregnancy Information**

- Early pregnancy signs and stages of fetal development.
- Importance of vitamins and healthy habits
- General pregnancy and childbirth-related information

1. **Breastfeeding**

- Information on duration (when to start and stop) and position
- Pain management
- Substitutes of breastfeed

## **Topic: Contraception and Family Planning:**

1. **Types of Contraceptives:**

- Oral contraceptives (pills)
- Male condoms
- Female condoms
- IUD (like Cu-T, hormonal IUD)
- Alternatives to existing methods

1. **Side Effects:**

- Temporary side effects
- Permanent side effects
- Home remedies to reduce side effects

1. **Usage Guidance:**

- Correct usage of male and female contraceptives
- Storage guidelines for contraceptives

1. **Effectiveness and Duration:**

- Duration
- Effectiveness of various contraceptives

1. **Family Planning Queries:**

- Effectiveness of methods
- Safety and benefits
- General information
- Family planning with financial instability
- Ways to make family planning methods accessible
- Ideal gap between children
- Timing considerations for having children
- How to approach family planning discussions
- Balancing personal circumstances with family planning goals

1. **Sterilization:**

- Female sterilization
- Vasectomy
- Types of sterilization methods

## **Topic: Sexual and Vaginal Health:**

1. **Sex-Related Queries:**

All queries in relation to sex and sexual health.

- Intercourse
- Communication Tips
- Sex toys/ masturbation/ lubricants
- Bleeding during/after intercourse
- Sexual health

1. **Vaginal Health and Discharge:**

- Vaginal discharge
- Itching and discomfort

1. **Sexually Transmitted Infections (STI/STD)**

- Types of STD/STI
- Medications and management of STI
- Information

1. **Urinary Tract Infections (UTI)**

- Pain and itching in the urine
- Medications
- Home remedies
- Symptoms and duration
- Causes and prognosis

1. **Vaginal/Uterine Infections**

- Abnormal discharges with lower abdominal pain
- Cyst in the uterus
- Pelvic pain and fever
- Swelling

1. **Reproductive Anatomy**

- Male body parts
- Female body parts
- Function and physiology of the reproductive system
- Sperms and eggs
- Fertilisation

## **Topic: HIV**

1. **Prevention**

- What measures can be taken to prevent HIV

1. **Symptoms and Early Detection**

- What are the symptoms, and how can you detect HIV in the early stages? What are the options available in India?

1. **Treatment**

- Treatment options available in India

1. **Stigma and Awareness**

## **Topic: Mental Health and Wellness**

1. **Information and Safety Concerns:**

- Work overload affecting health
- General information on mental health causes and effects
- Anxiety and stress
- Tiredness
- Sleep problems or insomnia due to stress
- Irritability and mood change

1. **Stress Management**

- Meditation
- Yoga
- Breathing exercises
- Doctor or therapist-related queries
- Medication for stress

## **Topic: Menstrual Health:**

1. **Menstrual Cycle Information: Duration of periods**

- Spotting
- General information related to periods
- Diet
- Typical length of the menstrual cycle
- Causes of irregular periods
- Treatments for irregular periods
- Medications to induce menstruation

1. **Period Pain Management**

- Home remedies (e.g., hot water bottles, yoga, exercise)
- Causes
- Pain relief medications

1. **Sanitary Products and Hygiene**

- Type of sanitary products
- Use of sanitary products
- Intimate wash and wipes, e.g., vaginal wash
- Undergarments
- Cleaning supplies: soaps, powders, and sprays

1. **Menstrual Flow**

- Abnormal flow
- Amount of bleeding

## **Topic: PCOS/PCOD:**

1. **Information:**

- General details about PCOS/PCOD.
- Commonly affects women of reproductive age.
- Caused by hormonal imbalances and genetics.
- Related to insulin resistance and high androgen levels.

1. **Symptoms:**

- Irregular periods or no periods at all.
- Excessive hair growth (hirsutism) on the face or body.
- Acne and oily skin.
- Weight gain or difficulty losing weight.
- Hair thinning or scalp hair loss.
- Fertility challenges due to lack of ovulation.

1. **Management:**

- Hormonal treatments (e.g., birth control pills).
- Insulin-sensitizing drugs (e.g., metformin).
- Healthy diet for weight management.
- Regular physical activity (e.g., cardio, strength training).
- Stress management through yoga or meditation.
- Natural remedies like herbal supplements.
- Regular check-ups with a healthcare provider.

## **Topic: Other:**

1. **Child health**

- Child vaccination, nutrition, height, weight, diarrhoea, ORS, common cold and ailments, hygiene

1. **Cultural, Religious, or Moral Norms**

- Queries or statements reflecting cultural, religious, or moral beliefs, restrictions, or taboos around SRH topics. These include doubts influenced by traditional or societal norms.

1. **Marriage and Relationships:**

- Queries concerning emotional, physical, or social aspects of romantic partnerships and marriages, especially in the context of SRH.

1. **Misconceptions and Myths**

- Queries that reflect incorrect or misunderstood beliefs about sexual, reproductive, or general health. Annotate here if the user’s question suggests misinformation or seeks clarification of myths.

1. **Health Equity and Access**

- Queries that highlight barriers or facilitators to accessing healthcare, medicines, or information, especially in resource-limited or rural contexts. (access to doctors or nurses, availability of vaccines, condoms, or other things)

1. **General Health Queries**

- Any other questions related to health that do not come under the categories mentioned above.

1. **Exercise and Fitness:**

- Types of exercises (yoga, strength training, Pilates)
- Impact on health (hormone balance, weight loss, period relief)
- Exercise frequency and home-based workouts

1. **Diet and Nutrition:**

- Best foods, snacks, and drinks (e.g., herbal teas, ginger chai)
- Dietary practices for health, hormonal balance, and weight management
- Healthy recipes and diet plans for general wellness and period relief
