# Supplementary material for: Understanding User Intent in Code-Mixed Sexual and Reproductive Health Queries in Urban India: Hierarchical Classification Approach Using Large Language Models
Source: J Med Internet Res. 2026 Mar 24;28:e86545. doi: 10.2196/86545 (PMC13012004; doi:10.2196/86545)
Supplement: Multimedia Appendix 2 [file jmir-v28-e86545-s002.docx]

LLM Prompting Details

For evaluating LLMs, we utilized the structured prompt template described in Table S1. Experiments were zero-shot and single-turn conversations (no conversation history or in-context examples). To ensure consistency and reproducibility across all LLM-based experiments, we set the temperature to 0.1, emphasizing deterministic and reliable outputs. We parsed each response against the predefined hierarchy and marked it *NotValid* only if the predicted Topic was not in the hierarchy. As this is a hierarchical classification task, if the predicted Topic is incorrect, the Subtopic is automatically counted as incorrect. Although the zero-shot prompt requested a model confidence score (0.0–1.0), this information was collected solely for potential future analysis and was not used in the current evaluation of model performance.

Table S1: Template for zero-shot prompting

Classify the following Hinglish (Romanized Hindi + English) query into exactly ONE topic and ONE subtopic from the intent hierarchy below.

### Intent Hierarchy:

{json.dumps(hierarchy, indent=4)}

### Query (Hinglish):

"{query}"

###Output Format:

Return your answer inside a JSON code block like this:

```json

{{

"Topic": "<selected_topic>",

"Subtopic": "<selected_subtopic_from_that_topic>",

"Confidence": <number between 0.0 and 1.0>,

"Reason": "<short reason>"

}}

```

### Rules:

1. Select ONLY ONE topic and ONE subtopic.
2. The subtopic MUST belongs to the selected topic.
3. Confidence MUST be a decimal number between 0.0 and 1.0.
4. Reason MUST be a short sentence (max 20 words).
5. Output MUST be valid JSON inside a JSON code block.

### Final instruction

Return just the json object in markdown format. Do not include any other text in the response.

Table S2: 95% confidence intervals (CIs) for zero-shot hierarchical classification performance across open-weight, Indic, and proprietary LLMs. In each category, the best performing model is highlighted in **bold**, and the second-best is underlined.

| Models | #Params | hF1  (95%CI) | Exact Match (95%CI) | Accuracy@l1 (95%CI) | Accuracy@l2 (95%CI) |
| --- | --- | --- | --- | --- | --- |
| Mixtral-8x7B-Instruct | 7B | 0.593  *(0.581–0.606)* | 0.453  *(0.439–0.469)* | 0.733  *(0.720–0.747)* | 0.617  *(0.600–0.635)* |
| Llama-3.1-8B-Instruct | 8B | 0.630  *(0.618–0.642)* | 0.491  *(0.476–0.506)* | 0.769  *(0.756–0.782)* | 0.638  *(0.622–0.655)* |
| Qwen-2.5-7b-Instruct | 7B | 0.605  *(0.593–0.618)* | 0.463  *(0.448–0.478)* | 0.747  *(0.735–0.761)* | 0.619  *(0.602–0.636)* |
| Aya-Expanse-8B | 8B | 0.528  *(0.515–0.542)* | 0.411  *(0.396–0.426)* | 0.646  *(0.631–0.660)* | 0.636  *(0.618–0.654)* |
| Gemma-2-9B-IT | 9B | 0.657  *(0.644–0.670)* | 0.544  *(0.528–0.559)* | 0.770  *(0.757–0.783)* | 0.706  *(0.691–0.722)* |
| Gemma-3-27B-IT | 27B | 0.739  *(0.728–0.750)* | 0.629  *(0.615–0.644)* | 0.849  *(0.838–0.859)* | 0.741  *(0.727–0.756)* |
| Llama-3.3-70B-Instruct | 70B | **0.742**  (0.731–0.753) | **0.630**  *(0.616–0.645)* | **0.853**  *(0.843–0.864)* | **0.738**  *(0.724–0.753)* |
| Airavata | 7B | 0.404  *(0.392–0.416)* | 0.226  *(0.213–0.239)* | 0.581  *(0.566–0.596)* | 0.389  *(0.369–0.408)* |
| Llama-3-Gaja-Hindi-8B | 8B | 0.596  *(0.584–0.609)* | 0.452  *(0.437–0.467)* | 0.740  *(0.727–0.754)* | 0.610  *(0.593–0.627)* |
| AryaBhatta | 8.5B | 0.365  *(0.354–0.376)* | 0.157  *(0.146–0.168)* | 0.574  *(0.559–0.589)* | 0.273  *(0.255–0.291)* |
| Krutrim-2-Instruct | 12B | 0.558  *(0.547–0.571)* | 0.386  *(0.371–0.401)* | 0.731  *(0.718–0.745)* | 0.527  *(0.510–0.546)* |
| Sarvam-M | 24B | **0.757**  *(0.746–0.768)* | **0.647**  *(0.633–0.662)* | **0.867**  *(0.856–0.877)* | **0.747**  *(0.732–0.761)* |
| GPT-5 | – | **0.784**  *(0.774–0.795)* | **0.683**  (0.669–0.697) | **0.886**  *(0.876–0.895)* | **0.771**  *(0.758–0.785)* |
| GPT-4o | – | 0.779  *(0.768–0.789)* | 0.675  *(0.660–0.689)* | 0.882  *(0.872–0.892)* | 0.764  *(0.751–0.778)* |
| Claude-3.5-Sonnet | – | 0.745  *(0.734–0.757)* | 0.639  *(0.625–0.654)* | 0.851  *(0.840–0.862)* | 0.751  *(0.736–0.765)* |

Table S3: Comparison of GPT-5 and GPT-4o on representative user queries.

| Query  (Hinglish ® English) | Ground Truth  (Topic® SubTopic) | GPT-5 | GPT-4o |
| --- | --- | --- | --- |
| safaiya kaise karwate hain?  *(How is cleaning done? )* | Pregnancy and PNC® Abortion | ✓ | 🗶 |
| Pregnancy me 5 month me vomiting hoti h to kya kre  *(What should I do if I am vomiting in the 5th month of pregnancy?)* | Pregnancy and PNC ® Antepartum | ✓ | 🗶 |
| Family planning Muslim community me accept hain kya?  *(Is family planning accepted in the Muslim community?)* | Contraception and Family Planning ® Family Planning Queries | 🗶 | 🗶 |
| Masik pali aane se Bhagvan ke pas kau nahi jana chahiye ?  *(Why should one avoid going to god/temples during periods?)* | Other ® Cultural, Religious, or Moral Norms | ✓ | ✓ |
| 1 sal se bacha rukhne ke liye try kar rahe hai lekin nahi rukh raha hai to kya karna padega?  (*We have been trying to have a child for one year, but it has not happened. What should we do?*) | Pregnancy and PNC ® Infertility | ✓ | ✓ |
| Family planning may agar koi mahila test tub karvati hai to kya hota hai ?  *(In family planning, if a woman undergoes test tube, what happens ? )* | Pregnancy and PNC ® Infertility | 🗶 | 🗶 |
| Kitni der jinda rahte hain sperm?  *(How long do sperm stay alive?)* | Sexual and Vaginal Health ® Reproductive Anatomy | 🗶 | 🗶 |
