# Supplementary material for: Understanding User Intent in Code-Mixed Sexual and Reproductive Health Queries in Urban India: Hierarchical Classification Approach Using Large Language Models
Source: J Med Internet Res. 2026 Mar 24;28:e86545. doi: 10.2196/86545 (PMC13012004; doi:10.2196/86545)
Supplement: Multimedia Appendix 3 [file jmir-v28-e86545-s003.docx]

Table S4: Model-generated reason for Error Type C1: Lexical Ambiguity and Euphemisms

| **Query**  **(Hinglish → English)** | **Model** | **Predicted**  **Topic → Subtopic** | **Model-Generated Reason** |
| --- | --- | --- | --- |
| safaiya kaise karwate hain?  *(How is an abortion done? )* | GPT-5 | Pregnancy & PNC → Abortion | “In context, *‘safai karwana’* commonly refers to abortion / D&C procedure guidance.” |
|  | Claude-3.5-Sonnet | Pregnancy & PNC → Abortion | “The query asks about *‘safaiya’*, which is a euphemism for abortion in Hindi.” |
|  | Llama-3.3-70B-Instruct | Menstrual Health → Sanitary Products and Hygiene | “*Safaiya* means sanitary pad.” |
|  | Gemma-3-27B-IT | Sexual & Vaginal Health → Vaginal Health and Discharge | “The query asks about *‘safaiya’*, which refers to vaginal hygiene / cleaning.” |
|  | Sarvam-M | Other → General Health Queries | “Query about general cleaning practices.” |
|  | Llama-3-Gaja-Hindi-8B | Menstrual Health → Sanitary Products and Hygiene | “The query is about how to maintain hygiene during menstruation.” |

Table S5: Model-generated reason for Error Type C2: Misclassifying Stage in the Reproductive Health Journey

| **Query**  **(Hinglish → English)** | **Model** | **Predicted**  **Topic → Subtopic** | **Model-Generated Reason** |
| --- | --- | --- | --- |
| Pregnancy me 5 month me vomiting hoti h to kya kre  *(What should I do if I am vomiting in the 5th month of pregnancy?)* | GPT-5 | Pregnancy & PNC → Antepartum | “Vomiting at five months pregnant; asking what to do during pregnancy.” |
|  | Claude-3.5-Sonnet | Pregnancy & PNC → Antepartum | “Query specifically asks about vomiting during 5th month of pregnancy, which is an antepartum concern.” |
|  | Llama-3.3-70B-Instruct | Pregnancy & PNC → Pregnancy Information | “Query about pregnancy symptoms.” |
|  | Gemma-3-27B-IT | Pregnancy & PNC → Pregnancy Information | “The query explicitly mentions pregnancy and asks about vomiting during the 5th month.” |
|  | Sarvam-M | Pregnancy & PNC → Antepartum | “Query about vomiting during 5th month of pregnancy.” |
|  | Llama-3-Gaja-Hindi-8B | Pregnancy & PNC → Pregnancy Information | “The query is about pregnancy and specifically about vomiting during the 5th month of pregnancy.” |

Table S6: Model-generated reason for Error Type C3: Cultural and Religious Context Error

| **Query**  **(Hinglish → English)** | **Model** | **Predicted**  **Topic → Subtopic** | **Model-Generated Reason** |
| --- | --- | --- | --- |
| Family planning Muslim community me accept hain kya?  (Is family planning accepted in the Muslim community?) | GPT-5 | Other → Cultural, Religious, or Moral Norms | “Asks about religious acceptance of family planning in Muslims.” |
|  | Claude-3.5-Sonnet | Other → Cultural, Religious, or Moral Norms | “Query specifically asks about family planning acceptance in Muslim community, relating to religious norms.” |
|  | Llama-3.3-70B-Instruct | Contraception & Family Planning → Family Planning Queries | “Query about family planning.” |
|  | Gemma-3-27B-IT | Contraception & Family Planning → Family Planning Queries | “The query directly asks about family planning acceptance within a specific community.” |
|  | Sarvam-M | Other → Cultural, Religious, or Moral Norms | “Query about cultural/religious acceptance of family planning.” |
|  | Llama-3-Gaja-Hindi-8B | Contraception & Family Planning → Family Planning Queries | “The query is about the acceptance of family planning in the Muslim community…” |
| Masik pali aane se Bhagvan ke pas kau nahi jana chahiye ?  (Why should one avoid going to god/temples during periods?) | GPT-5 | Other → Cultural, Religious, or Moral Norms | “Asks about religious practice during menstruation, not medical information.” |
|  | Claude-3.5-Sonnet | Other → Cultural, Religious, or Moral Norms | “Query asks about religious restrictions during menstruation regarding visiting temples/religious places.” |
|  | Llama-3.3-70B-Instruct | Menstrual Health → Menstrual Cycle Information | “Query about menstrual cycle.” |
|  | Gemma-3-27B-IT | Other → Misconceptions and Myths | “The query relates to a superstitious belief about menstruation and religious practices.” |
|  | Sarvam-M | Other → Cultural, Religious, or Moral Norms | “Query about religious restrictions during menstruation.” |
|  | Llama-3-Gaja-Hindi-8B | Menstrual Health → Menstrual Cycle Information | “The query is about understanding the menstrual cycle and its relation to religious beliefs.” |

Table S7: Model-generated reason for Error Type C4: Misunderstanding Reproductive Intent

| **Query**  **(Hinglish → English)** | **Model** | **Predicted**  **Topic → Subtopic** | **Model-Generated Reason** |
| --- | --- | --- | --- |
| 1 sal se bacha rukhne ke liye try kar rahe hai lekin nahi rukh raha hai to kya karna padega?  (*We have been trying to have a child for one year, but it has not happened. What should we do?*) | GPT-5 | Pregnancy & PNC → Infertility | “Trying to conceive for a year without success; seeking guidance.” |
|  | Claude-3.5-Sonnet | Pregnancy & PNC → Infertility | “Query is about trying unsuccessfully to conceive a child for 1 year.” |
|  | Llama-3.3-70B-Instruct | Contraception & Family Planning → Family Planning Queries | “Query about preventing child.” |
|  | Gemma-3-27B-IT | Contraception & Family Planning → Family Planning Queries | “The query explicitly asks about preventing pregnancy for a year without success.” |
|  | Sarvam-M | Contraception & Family Planning → Family Planning Queries | “Query about inability to stop childbirth despite efforts aligns with family planning concerns.” |
|  | Llama-3-Gaja-Hindi-8B | Contraception & Family Planning → Effectiveness and Duration | “The query is about trying to delay pregnancy for a year and seeking guidance on what to do if it doesn't work.” |
| Family planning may agar koi mahila test tub karvati hai to kya hota hai ?  *(In family planning, if a woman undergoes test tube, what happens ? )* | GPT-5 | Contraception & Family Planning → Sterilization | “Asking what happens when a woman gets tubal sterilization for family planning.” |
|  | Claude-3.5-Sonnet | Contraception & Family Planning → Family Planning Queries | “Query specifically asks about family planning tests for women in Agra.” |
|  | Llama-3.3-70B-Instruct | Contraception & Family Planning → Sterilization | “Mentions test tub.” |
|  | Gemma-3-27B | Contraception & Family Planning → Family Planning Queries | “The query directly asks about a procedure related to family planning (test tube baby).” |
|  | Sarvam-M | Contraception & Family Planning → Sterilization | “Query about tubal ligation (test tub karvati) for family planning.” |
|  | Llama-3-Gaja-Hindi | Contraception & Family Planning → Family Planning Queries | “The query is about a woman's family planning and testing for tubal blockage.” |

Table S8: Model-generated reason for Error Type C5: Misclassifying Questions about Bodily Anatomy or Processes

| **Query**  **(Hinglish → English)** | **Model** | **Predicted**  **Topic → Subtopic** | **Model-Generated Reason** |
| --- | --- | --- | --- |
| Kitni der jinda rahte hain sperm?  (How long do sperm stay alive?) | GPT-5 | Sexual & Vaginal Health → Sex-Related Queries | “Asks about how long sperm survive, a sex-related query.” |
|  | Claude-3.5-Sonnet | Sexual & Vaginal Health → Reproductive Anatomy | “Query asks about sperm survival duration, which relates to reproductive anatomy and function.” |
|  | Llama-3.3-70B-Instruct | Contraception & Family Planning → Types of Contraceptives | “Query about sperm lifespan.” |
|  | Gemma-3-27B | Contraception & Family Planning → Effectiveness and Duration | “The query asks about the lifespan/duration of ‘ispram’ (spermicide), relating to its effectiveness.” |
|  | Sarvam-M | Other → General Health Queries | “Inquiry about lifespan duration without context to specific medical conditions.” |
|  | Llama-3-Gaja-Hindi | Pregnancy & PNC → Pregnancy Information | “The query is asking about the duration of pregnancy…” |
